# Exhaled Carbon Monoxide Indicates Persistent Subgingival Dysbiosis After Periodontal Therapy

**DOI:** 10.1111/jcpe.70053

**Published:** 2025-10-16

**Authors:** Sophie Koehlen, Inga Harks, Johannes Matern, Karola Prior, Peter Eickholz, Katrin Lorenz, Ti‐Sun Kim, Thomas Kocher, Jörg Meyle, Doğan Kaner, Yvonne Jockel‐Schneider, Dag Harmsen, Benjamin Ehmke, Sven Kleine Bardenhorst, Daniel Hagenfeld

**Affiliations:** ^1^ Department of Periodontology and Operative Dentistry Münster University Hospital Münster Germany; ^2^ Department of Periodontology, Center for Dentistry and Oral Medicine (Carolinum) Goethe University Frankfurt am Main Frankfurt am Main Germany; ^3^ Policlinic of Operative Dentistry, Periodontology, and Pediatric Dentistry, Division of Periodontology, Faculty of Medicine, University Hospital Carl Gustav Carus Dresden University of Technology Dresden Germany; ^4^ Section of Periodontology, Department of Conservative Dentistry, Clinic for Oral, Dental and Maxillofacial Diseases Heidelberg University Hospital Heidelberg Germany; ^5^ Department of Restorative Dentistry, Periodontology and Endodontology University Medicine Greifswald Greifswald Germany; ^6^ Department of Periodontology University of Giessen Giessen Germany; ^7^ Departments of Periodontology and Synoptic Dentistry Charite‐Universitätsmedizin Berlin Germany; ^8^ Department of Periodontology, Dental School, Faculty of Health University of Witten/Herdecke Witten Germany; ^9^ Department of Periodontology University Hospital Würzburg Würzburg Germany; ^10^ Institute of Epidemiology and Social Medicine University of Münster Münster Germany

**Keywords:** 16S rRNA gene sequencing, carbon monoxide, dysbiosis, periodontitis, smoking, subgingival microbiome

## Abstract

**Aim:**

To investigate whether exhaled carbon monoxide (CO) is dose‐dependently associated with subgingival polymicrobial clusters before and after non‐surgical periodontal therapy.

**Material and Methods:**

We followed 163 adults with periodontitis, measuring CO before and at 2, 14 and 26 months after therapy. Subgingival plaque samples were analysed using 16S rRNA gene sequencing. We applied topic models and regression analysis, adjusted for age, sex, treatment group, pocket probing depth and plaque levels.

**Results:**

Smokers had generally higher mean CO levels at every visit and showed significant declines at 14 and 26 months after therapy (*p* < 0.01). Before therapy, CO was not associated with dysbiosis; after therapy, higher levels were positively associated. In sensitivity analyses, self‐reported smoking status explained more variance in dysbiosis than CO alone, with only marginal gains when both were combined. Among smokers, a distinct polymicrobial cluster dominated by *Fusobacterium* and *Prevotella* increased with higher exhaled CO. Every 10 parts per million increase in CO was associated with an estimated 16% increase in cluster abundance (95% confidence interval: 8%–26%).

**Conclusion:**

Exhaled CO may help monitor persistent dysbiosis and select an anaerobe‐dominated subgingival community after periodontal therapy among smokers.

## 1 Introduction

Periodontitis, one of the leading causes of tooth loss in adults, is characterised by the destruction of the tissues supporting the teeth (Pihlstrom et al. 2005). It is primarily driven by the host's immune response to a dysbiotic subgingival microbial community (Hajishengallis and Lamont 2021). Several behavioural and environmental aspects are known to influence the onset and progression of periodontitis, with smoking being one of the most significant factors (Papapanou et al. 2018). Smokers have consistently been shown to be at a higher risk of developing periodontitis and experience more severe disease progression compared to non‐smokers (Bergström et al. 2000; Calsina et al. 2002; Bergström 2003). Smoking not only affects the clinical parameters, such as pocket depth and attachment loss, but also exerts a profound influence on the composition of the subgingival microbiome (Coretti et al. 2017; Al Kawas et al. 2021).

Smokers with periodontitis tend to harbour a subgingival microbial profile associated with an increased pathogenic potential, including higher levels of periodontal pathobionts such as *Porphyromonas gingivalis* and *Tannerella forsythia* , as well as the genus *Fusobacterium* (Bizzarro et al. 2013; Haffajee and Socransky 2001; Tamashiro et al. 2023). These changes in microbial composition contribute to the heightened risk and severity of periodontitis in smokers, potentially due to the immunomodulatory effects of cigarette smoke on the host's immune response (Lee et al. 2012).

Carbon monoxide (CO) is a colourless, odourless gas that is toxic at high concentrations yet is produced endogenously via haem oxygenase–mediated haem degradation and in small amounts by microorganisms (Katayama et al. 2024). In the oral cavity, CO can occur from external sources (e.g., tobacco smoke) as well as endogenously through tissue metabolism (Coburn et al. 1963). It remains unclear whether oral bacteria actively metabolise CO, using it as an energy source, enzymatically converting or detoxifying it, and whether exhaled CO can serve as an indicator of subgingival dysbiosis in both smokers and non‐smokers during periodontal therapy.

Therefore, we sought to investigate the dose‐dependent effects of CO on the subgingival microbiome in smoking and non‐smoking patients with periodontitis receiving non‐surgical periodontal treatment (NSPT) with a 2‐year follow‐up. Using a Smokerlyzer device, we aimed to objectively measure exhaled CO and analyse its impact on the microbial composition.

## 2 Materials and Methods

### 2.1 Study Characteristics

#### 2.1.1 Study Design

The current study is a secondary analysis of the data derived from the ABPARO trial (ISRCTN: 64254080, ClinicalTrials.gov: NCT00707369), a multicentre, randomised, double‐blind, placebo‐controlled, parallel‐group, phase IV efficacy study (Eickholz et al. 2016, 2019, 2023; Harks et al. 2014, 2015). Conducted across eight university hospitals in Germany, the study was designed to evaluate the long‐term effects of adjunctive antibiotics on non‐surgical periodontal treatment (NSPT). Each patient was involved in the trial for an average of 27.5 months. The study protocol was approved by the Medical Ethics Committee of the University of Münster (ref: 2016–505‐f‐S). The ABPARO trial enrolled 345 per‐protocol patients aged 18–75 years who were diagnosed with untreated localised to generalised stage III‐IV periodontitis. Detailed inclusion criteria are available (Harks et al. 2014, 2015). Periodontal measurements and plaque sampling began at baseline (V2), followed by an additional visit for periodontal therapy (V3), which involved full‐mouth NSPT and the administration of blinded medication. Patients received a combination of amoxicillin and metronidazole (amoxicillin 3H_2_O 574 mg [Amoxicillin‐Ratiopharm 500 mg, Ratiopharm, Germany], metronidazole 400 mg [Flagyl 400, Sanofi‐Aventis, Germany]) or a placebo three times daily for 7 days. Re‐evaluation and plaque sampling took place at V4, which occurred 2 months after NSPT, and follow‐up visits with subgingival plaque sampling were conducted at V6 (8 months post NSPT), V8 (14 months post‐NSPT) and V12 (26 months post‐NSPT). Supportive periodontal therapy, consisting of subgingival and supragingival debridement as part of a standardised full‐mouth mechanical debridement protocol using ultrasonic scalers, hand instruments and air powder, was provided at 3‐month intervals from V5 through V12. Smoking status was assessed via a questionnaire, and CO measurements were performed using the Bedfont Micro 4 Smokerlyzer (Bedfont, UK) on baseline visit (V2), re‐evaluation (V4) and 14 and 26 months (V8, V12) after NSPT. The O'Leary plaque index (O'Leary et al. 1972) was used for monitoring oral hygiene, and instructions were given on baseline visit (V2) and at 3‐month intervals from V4 through V12. Smoking cessation counselling was not part of the study protocol (Harks et al. 2014).

#### 2.1.2 Patient Sub‐Sample

For this secondary analysis, a sub‐sample of 163 subjects randomly drawn from the per‐protocol stratum was analysed. Of those, 69 were identified as smokers while 94 were non‐smokers by questionnaire (Hagenfeld et al. 2020).

#### 2.1.3 Plaque Sampling

Subgingival plaque samples were collected from four teeth (one per quadrant) in each participant as described previously (Harks et al. 2016). Briefly, sample teeth were selected based on a randomised clockwise or counterclockwise sequence, starting in one quadrant and proceeding systematically through the dentition. In each quadrant, the first tooth meeting predefined criteria (PPD ≥ 6 mm) was selected, continuing through up to four full rounds until four eligible teeth were identified. Supragingival plaque was first removed with a cotton swab, and then sterile paper points were inserted into the periodontal pockets for 10 s to collect subgingival plaque. These paper points were pooled into a single sterile collection tube and stored at −20°C until further analysis, as previously described (Hagenfeld et al. 2018, 2019).

#### 2.1.4 Microbiome Analysis

Microbiome analysis was conducted by extracting bacterial genomic DNA from the plaque samples using the QIAamp Mini DNA Isolation Kit (Qiagen, Hilden, Germany). The DNA was purified, and the V4 hypervariable region of the bacterial 16S rRNA gene was amplified with eubacterial and barcode primers carrying out two polymerase chain reactions according to the manufacturer's protocol with minor modifications, as described in detail in our previous publications (Hagenfeld et al. 2018, 2019). Up to 96 libraries supplemented by a mock sample and negative controls were pooled and sequenced in Illumina MiSeq runs using 250 base‐pair paired‐end reads (MiSeq Reagent Kit Version 2, Illumina). All raw reads are available from the European Nucleotide Archive (http://www.ebi.ac.uk/ena/) under study accession number PRJEB51017. Illumina's MiSeq control software v.2.6.2.1, real‐time analysis v.1.18.54, MiSeq reporter v.2.6.3 and Cutadapt v.1.8.1 were used for initial data processing, as described previously (Hagenfeld et al. 2018, 2020; Martin 2011). Adapter‐ and primer‐free FastQ files were further analysed using R v.4.0.4 and RStudio v.1.1.463 (R Development Core Team 2017; RStudio Team 2016), with the DADA2 pipeline v.1.20.0 (Callahan et al. 2016). Reads were trimmed to remove low‐quality bases at both ends, and a maximum of two expected errors per read was allowed to filter low‐quality sequences. Ribosomal sequence variants (RSVs) were assigned taxonomically using a naïve Bayesian classifier with the expanded Human Oral Microbiome Database (eHOMD) v.15.1 (Chen et al. 2010). RSVs appearing in fewer than three samples and with an abundance below 100 reads were removed. Phyloseq v.1.36.0 was used to combine data from all runs, and run quality was validated as per previous quality control protocols (Hagenfeld et al. 2023; McMurdie and Holmes 2013).

#### 2.1.5 CO Measurement

CO levels were measured using the Bedfont Micro 4 Smokerlyzer, following the manufacturer's instructions. Subjects were instructed to inhale deeply for 15 s, hold their breath and then exhale slowly and fully into the Smokerlyzer mouthpiece. CO concentrations were recorded in parts per million (ppm) (Harks et al. 2014).

### 2.2 Statistical Analysis

Statistical significance was determined using an alpha level of 0.05. To test differences in the distributions of the CO value at baseline, the Wilcoxon rank‐sum test was used for analysis of two groups, and the Kruskal–Wallis test was used for analysis of more than two groups. For the changes in CO value over time, the Wilcoxon signed‐rank test was used to account for the paired nature of the data. The primary endpoint of this study was the association between exhaled CO levels and subgingival microbial dysbiosis following non‐surgical therapy. A generalised linear model was constructed to examine the influence of CO measurements on subgingival microbial dysbiosis (as quantified in Kleine Bardenhorst et al. 2024) while controlling for the confounding factors age, sex and treatment group. The latter analysis was stratified by timepoint to account for temporal changes in the effects of CO values on microbial composition. To assess the robustness of our findings regarding the influence of CO measurements versus baseline self‐reported smoking status on dysbiosis, we performed a series of sensitivity analyses at each study visit (V2, V4, V8, V12). For each visit, we fitted three linear regression models, all adjusted for age, sex and treatment group: (i) Model A (CO only): Dysbiosis ~ CO + age + sex + treatment group; (ii) Model B (Smoking only): Dysbiosis ~ smoking status + age + sex + treatment group; (iii) Model C (Combined): Dysbiosis ~ CO + smoking status + age + sex + treatment group. We compared model fit using the Akaike information criterion (AIC) and explained variance (*R* ^2^). Nested *F*‐tests (via ANOVA) assessed whether adding smoking status to the CO‐only model significantly improved the fit. Variance‐inflation factors (VIFs) for Model C were computed to screen for collinearity between CO and self‐reported smoking. To better understand the collective microbial response to exhaled CO exposure in smokers, we developed a polymicrobial marker using latent Dirichlet allocation (LDA), as described recently (Saberi Kakhki et al. 2025), to identify polymicrobial clusters that co‐vary with CO levels. This approach addresses the limitation of examining individual taxa in isolation and provides a more comprehensive view of community‐level changes. A zero‐inflated negative binomial mixed‐effects model was used to examine associations between CO levels and the polymicrobial marker abundance across the 26‐month follow‐up period, incorporating subject‐specific random intercepts and adjusting for time‐varying confounders including periodontal treatment status, plaque scores, pocket probing depth and antibiotic use.

## 3 Results

### 3.1 Analysis at Baseline Visit

Table 1 summarises the baseline exhaled CO measurements (in ppm). As expected, self‐reported smoking status was a strong indicator, with smokers showing considerably elevated CO values relative to non‐smokers. CO readings did vary with age, with older groups exhibiting progressively lower measurements. Individuals classified as grade C had notably higher mean CO values than those in grade B. Furthermore, differences were observed between baseline quantiles of pocket depth and plaque measures, although without a clear gradient and with high variability across quantiles. Baseline CO measures stratified by smoking status are shown in Table S1.

**TABLE 1:** Baseline variables in relation to the CO measurements at the baseline visit.

Variable	*n* (total = 163)	Mean (SD)	Median (IQR)	Min.	Max.	*p‐value*
Smoking status						
Non‐Smoker	94	0.69 ± 1.13	0.00 (1.00)	0.00	6.00	**< 0.01**
Smoker	69 (NA = 0)	17.06 ± 8.53	15.00 (9.00)	7.00	51.00	
Centre						
Berlin	21	9.29 ± 12.76	3.00 (14.00)	0.00	51.00	0.876
Dresden	8	3.12 ± 4.73	0.50 (4.50)	0.00	12.00	
Frankfurt	12	7.08 ± 7.04	6.00 (12.50)	0.00	17.00	
Giessen	13	8.54 ± 11.06	3.00 (9.00)	0.00	37.00	
Greifswald	11	7.09 ± 8.01	7.00 (9.50)	0.00	21.00	
Heidelberg	10	5.90 ± 5.51	6.00 (10.75)	0.00	12.00	
Münster	51	7.61 ± 10.93	1.00 (12.50)	0.00	42.00	
Würzburg	37 (NA = 0)	8.14 ± 9.32	2.00 (16.00)	0.00	31.00	
Treatment group						
Antibiotic	81	7.91 ± 10.49	2.00 (12.00)	0.00	51.00	0.713
Placebo	82 (NA = 0)	7.33 ± 9.23	1.00 (13.00)	0.00	36.00	
Sex						
Female	83	8.13 ± 9.98	3.00 (13.00)	0.00	51.00	0.551
Male	80 (NA = 0)	7.09 ± 9.75	1.00 (12.00)	0.00	42.00	
Age, years						
< 45	36	13.17 ± 11.30	11.50 (17.25)	0.00	42.00	**< 0.01**
45–55	65	8.65 ± 8.95	6.00 (15.00)	0.00	37.00	
> 55	62 (NA = 0)	3.32 ± 7.90	0.00 (1.00)	0.00	51.00	
Stage						
III localised	20	4.65 ± 6.80	1.00 (7.00)	0.00	22.00	0.653
III generalised	90	8.20 ± 10.83	2.00 (14.50)	0.00	51.00	
IV	53 (NA = 0)	7.75 ± 8.99	2.00 (13.00)	0.00	42.00	
Grade						
B	25	3.16 ± 5.70	1.00 (3.00)	0.00	22.00	**< 0.01**
C	62 (NA = 76)	9.74 ± 9.29	10.00 (16.75)	0.00	37.00	
O'Leary category						
Q1 (0.00% to ≤ 17.59%)	41	10.56 ± 12.20	8.00 (19.00)	0.00	51.00	**< 0.02**
Q2 (> 17.59% to ≤ 33.62%)	41	4.76 ± 6.73	1.00 (11.00)	0.00	22.00	
Q3 (> 33.62% to ≤ 52.88%)	41	9.27 ± 9.51	9.00 (16.00)	0.00	42.00	
Q4 (> 52.88% to ≤ 100.00%)	40 (NA = 0)	5.85 ± 9.36	1.00 (11.00)	0.00	37.00	
Amount PPD ≥ 5 mm % category						
Q1 (3.00% to ≤ 11.00%)	41	8.17 ± 12.10	1.00 (12.00)	0.00	51.00	0.293
Q2 (> 11.00% to ≤ 18.00%)	41	5.95 ± 7.94	2.00 (11.00)	0.00	32.00	
Q3 (> 18.00% to ≤ 28.00%)	41	6.93 ± 9.95	1.00 (12.00)	0.00	42.00	
Q4 (> 28.00% to ≤ 78.00%)	40 (NA = 0)	9.48 ± 8.92	9.50 (16.25)	0.00	36.00	

*Note:* Smoking status was assessed via self‐report (questionnaire); Centre refers to study centre in which the treatment and data collection was performed; Stage refers to periodontitis stage according to the 2018 classification; Grade refers to periodontitis grade according to the 2018 classification; O'Leary refers to the O'Leary supragingival plaque index; Amount of PPD ≥ 5 mm refers to the % of sites with pocket probing depths of 5 mm or more; *n*, number of subjects in the group; Q1–4, quantiles 1–4; NA, not available values (missings); mean: mean CO measurement (in ppm) of the grouping variable at baseline visit; SD, standard deviation; median, median of the CO measurements (in ppm) of the group at baseline visit; IQR, interquartile range; Min., minimum CO measurement (in ppm) of the confounder group at baseline visit; Max., maximum CO measurement (in ppm) of the confounder group at baseline visit; *p*‐value, tested using Kruskal–Wallis rank‐sum test/Mann–Whitney *U* test and considered statistically significant if < 0.05.

### 3.2 Missing Data Analysis

While CO measurements were available for all participants at baseline, there were missing data at later timepoints. Specifically, there were four missing CO measurements at V4 (re‐evaluation, 2 months after NSPT) and three missing measurements at V12 (24 months after re‐evaluation, 26 months after NSPT). Of these, three missing values were from non‐smokers, while the remaining four were from smokers. Importantly, none of the participants had missing CO measurements at multiple timepoints, and the pattern of missing data appeared random. Therefore, for our pairwise tests, we used only complete cases.

### 3.3 Analysis of CO Over Time

**FIGURE 1:**
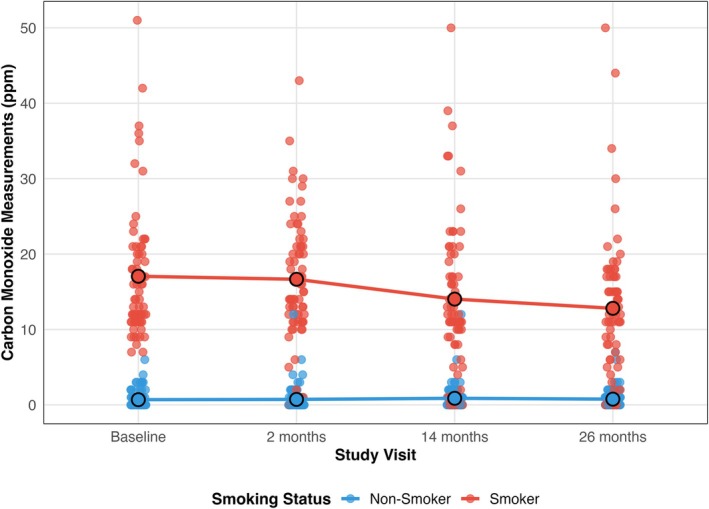
Carbon monoxide (CO) measurements (ppm) across study visits for smokers (red) and non‐smokers (blue). Individual data points are shown jittered for clarity, with solid lines and larger points indicating the group‐mean at each visit.

In smokers, significant decreases in CO levels were detected from baseline to 14 months (*p* < 0.01) and baseline to 26 months after NSPT (*p* < 0.01) (Figure 1). In contrast, no statistically significant changes in CO measurements were observed in non‐smokers across all the study visits.

### 3.4 Linear Model of the Effects of CO on Subgingival Dysbiosis

**FIGURE 2:**
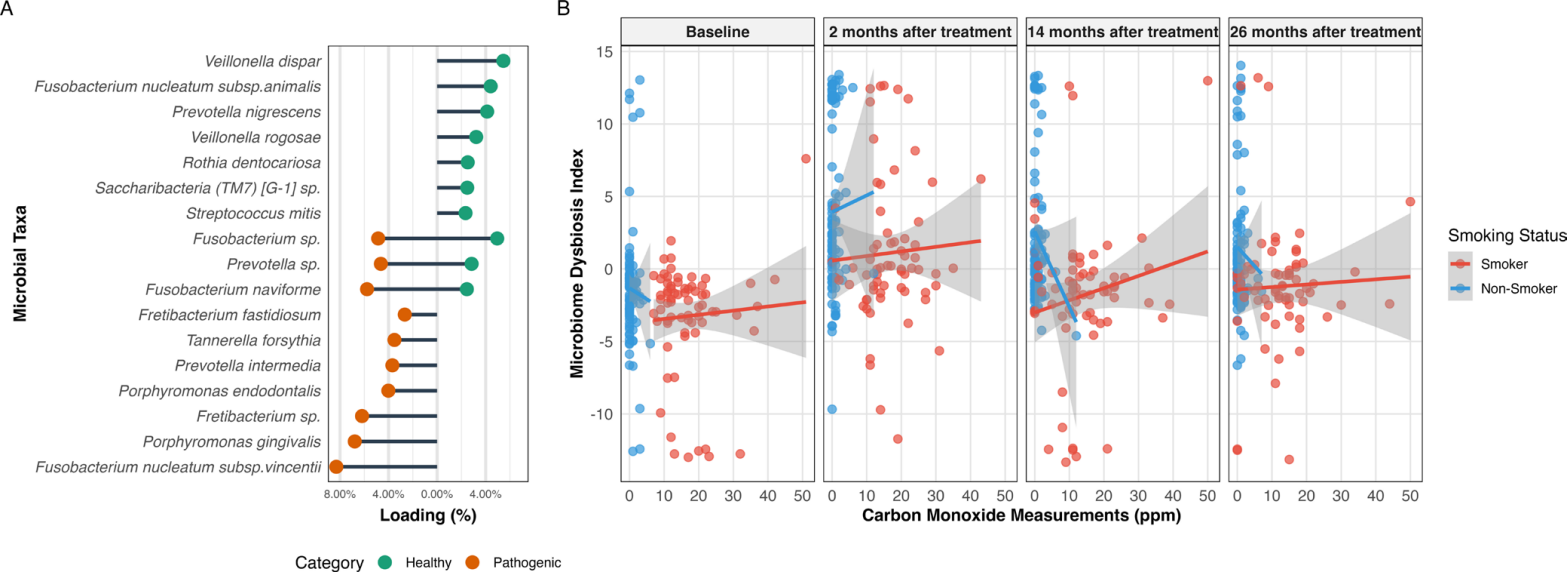
Association between exhaled carbon monoxide (CO) and subgingival microbiome dysbiosis. (A) Microbial taxa contributing most strongly to the dysbiosis index, with loading percentages indicating their relative influence. Taxa are categorised as health‐associated (green) or pathogenic (orange). (B) Scatterplots showing the relationship between CO levels (ppm) and the microbiome dysbiosis index at baseline and three follow‐up timepoints after treatment. Data are stratified by self‐reported smoking status (smokers: red; non‐smokers: blue), with linear fits and 95% confidence intervals shown.

Figure 2 illustrates the linear relationship between exhaled CO levels (in ppm) and dysbiosis as described previously (Kleine Bardenhorst et al. 2024) across all participants and timepoints, with points coloured by smoking status. Non‐smokers clustered at lower CO values and correspondingly lower dysbiosis scores, whereas smokers tended to exhibit higher CO levels and a broader range of dysbiosis.

The linear regression adjusted for treatment group (only at timepoints after treatment), age and sex stratified by timepoints revealed that, before periodontal therapy, there was no significant association between CO measurements and dysbiosis (*β* = 0.06; 95% CI: –0.01 to –0.13; *p* = 0.090) (Table S2). However, after NSPT, CO levels were positively associated with dysbiosis. This effect persisted up to 26 months after treatment (*β* = 0.09; 95% CI: 0.01–0.17; *p* = 0.025).

### 3.5 Sensitivity Analysis of Self‐Reported Smoking Versus CO Measurement

Across all visits, Model B (smoking status alone) consistently achieved lower AIC and higher *R* ^2^ than Model A (CO only) (Table S3). Incorporating both predictors (Model C) yielded only marginal *R* ^2^ gains and mixed effects on AIC (improved at V4 and V12, but slightly worse at V2 and V8). Nested analyses of variance (ANOVAs) showed that adding smoking status to CO‐only models produced statistically significant improvements in fit at every visit (all *p* < 0.05), indicating that self‐reported smoking contributes unique information beyond CO measurements. Furthermore, on adding the smoking status to the linear model, the association was not statistically significant anymore, showing a high level of variance explained by self‐reported smoking status.

### 3.6 Stratified CO Analysis for Smokers

**FIGURE 3:**
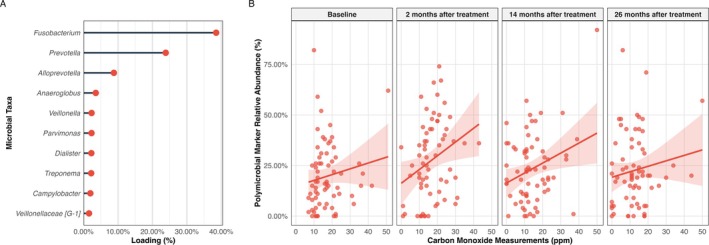
Association between exhaled carbon monoxide (CO) and polymicrobial marker abundance. (A) Microbial taxa contributing most strongly to the polymicrobial marker, with loading percentages reflecting their relative importance. (B) Scatterplots showing the relationship between CO levels (ppm) and the relative abundance of the polymicrobial marker at baseline and three follow‐up timepoints after treatment. Linear trends with 95% confidence intervals are shown across the full range of CO values.

We conducted a stratified analysis limited to self‐reported smokers to test whether variation in CO levels provides additional explanatory value beyond smoking status alone in modulating the subgingival ecosystem. LDA modelling identified a distinct polymicrobial cluster that co‐varied with exhaled CO levels specifically within the smoker cohort (Figure 3). This CO‐associated cluster showed consistent temporal patterns across the 26‐month follow‐up period, with sustained positive correlations between CO exposure and cluster abundance at all timepoints. The polymicrobial CO cluster was dominated by the anaerobic taxa *Fusobacterium* (~40% loading), *Prevotella* (~25% loading) and minor contributors. Zero‐inflated negative binomial mixed‐effects modelling revealed a significant association (*β* = 1.16; 95% CI: 1.08–1.26) per 10 ppm increase in CO and the polymicrobial marker abundance after adjusting for age, sex, PPD, plaque index and antibiotic treatment (Table S4).

## 4 Discussion

Our results suggest that exhaled CO levels are a supplementary indicator of subgingival dysbiosis in our mixed population of smokers and non‐smokers following NSPT. This is supported by the observation of a CO‐dependent polymicrobial cluster in smokers dominated by anaerobic *Fusobacterium* and *Prevotella*.

This study is unique in several aspects, including the sample size, the longitudinal design and the application of 16S rRNA gene sequencing for microbiome analysis. To our knowledge, no other studies have examined subgingival microbiome samples in periodontitis patients over a 2‐year period, particularly focusing on dose‐dependent effects of CO on subgingival microbial dysbiosis.

The identification of a distinct CO‐associated polymicrobial cluster dominated by anaerobic taxa provides new evidence for CO's role as an environmental selector in the subgingival microbiome. This is consistent with phylum‐level patterns reported in smokers, including depletion of Proteobacteria and enrichment of Firmicutes and Actinobacteria harbouring obligate anaerobic species (Wu et al. 2016). Our findings align with emerging understanding that CO functions as an ecological filter, altering the competitive ecosystem within periodontal pockets through multiple interconnected mechanisms (Mendes et al. 2021). The 250‐fold higher affinity of CO for haemoglobin compared to oxygen creates a cascade of environmental changes that favour the specific microbial cluster we identified (Rose et al. 2017). In periodontal pockets of smokers, this preferential binding results in relative tissue hypoxia, creating conditions where obligate anaerobes may outcompete their aerobic counterparts (Celik and Kantarci 2021). This hypoxic environment directly explains why our polymicrobial marker was dominated by strictly anaerobic genera such as *Fusobacterium* (~40% loading), *Prevotella* (~25% loading) and other oxygen‐intolerant species. Our results are supported by a cross‐sectional microbiome analysis of the subgingival periodontitis microbiome, which found *Fusobacterium*, *Fretibacterium*, *Streptococcus*, *Veillonella*, *Corynebacterium*, *TM7* and *Filifactor* more abundant in smokers compared to non‐smokers (Moon et al. 2015). *Prevotella* was significantly more abundant in salivary microbiomes of current smokers compared to non‐smokers (Wirth et al. 2020).

Furthermore, CO exerts direct inhibitory effects on aerobic bacteria through binding to haem and metalloenzymes in their respiratory chains (Wareham et al. 2015). This mechanism creates a selective pressure where CO concentrations that are subtoxic for anaerobic species become inhibitory for oxygen‐dependent commensals. The resulting microbial selection process may favour the CO‐tolerant polymicrobial cluster, even when controlled for the antibiotic treatment effect and reducing periodontal pockets (Figure 3). Additionally, the vasoconstrictor effects of nicotine, which was not part of this investigation, may amplify this selective pressure by reducing local blood flow and further decreasing oxygen tension in periodontal tissues (Song et al. 2017). Systemic CO binding and local vasoconstriction may create an increasingly hypoxic subgingival environment that progressively favours the anaerobic polymicrobial cluster we observed in our LDA analysis. The sensitivity analyses revealed that baseline self‐reported smoking status is a stronger and more parsimonious predictor of dysbiosis scores than exhaled CO measurements alone, indicating that CO and smoking status capture distinct facets of tobacco exposure.

Some non‐smokers in our study presented CO levels above 5 ppm. Possible explanations for these elevated levels include passive smoking, environmental exposure to CO and the consumption of certain foods that affect the hydrogen levels in exhaled air (Kotz 2012). The Bedfont Smokerlyzer, which we used in our study, suggests several cut‐off levels for distinguishing between non‐smokers, borderline smokers and smokers of varying levels of addiction (Harks et al. 2015). These cut‐off levels range from 0 to 6 ppm for non‐smokers to > 35 ppm for very heavy smokers. Other literature recommends cut‐offs ranging from 3 to 8 ppm (Jarvis et al. 1987; Javors et al. 2005; Middleton and Morice 2000). In our study, we followed established guidelines but recognised that variability in CO levels might still occur, particularly in low, passive or infrequent smokers, due to the short half‐life of CO. Similar findings were reported in another study, where some non‐smokers had elevated CO readings, and upon further questioning, some admitted to smoking after learning the significance of their CO levels (Middleton and Morice 2000). Additionally, this observation supports the speculation that oral bacteria could produce CO, artificially inflating measurements. Certain strains of the genera *Proteus* and *Morganella* can produce CO when cultured in media containing haem compounds, with production enhanced by the addition of haemin (Hayashi et al. 1985). Similarly, certain bacteria such as *Streptococcus mitis* —an alpha‐haemolytic species—and *Bacillus cereus* have been shown to produce CO from haem compounds via enzymatic degradation pathways under aerobic conditions but not anaerobically (Engel et al. 1972).

While our study has several strengths, there are also some limitations to consider. The relatively short half‐life of approximately 4.5 h may introduce bias, especially among infrequent smokers if the time of the last smoked cigarette is not considered (Sandberg et al. 2011). The gold standard for detecting smoking status is the measurement of cotinine in plasma, saliva or urine, which offers higher sensitivity and specificity compared to CO (Jarvis et al. 1987). However, for clinical purposes, CO measurement is more practical because of its non‐invasive nature, ease of use and rapid results. Although cotinine testing provides a more accurate assessment, the sensitivity (90%) and specificity (89%) of CO measurements at a cut‐off of 8 ppm are sufficient for most clinical and research applications (Jarvis et al. 1987). Furthermore, we have not enquired about the number of cigarettes smoked daily and for how many years the subjects had been smoking. Measurement errors due to Smokerlyzer calibration or environmental CO exposure (e.g., due to traffic emissions) could also affect accuracy. As we compared only a binary smoking variable and did not ask when the last cigarette was smoked, this may introduce variability in CO measurements, as we could not clearly associate smoking intensity with CO levels. Additionally, we were unable to reliably analyse the microbiome at the species level because of our short read sequencing approach. This limited resolution may have masked species‐specific associations with dysbiosis or CO levels. Further studies should include comparative trials with smokers undergoing regular CO monitoring, a detailed questionnaire on pack‐years and time since the last cigarette, alongside smoking cessation programs, to evaluate the long‐term effects of CO monitoring on smoking behaviour. Longitudinal data, combined with microbiome analysis, could provide deeper insights into how smoking cessation impacts the subgingival microbiome over time.

## 5 Conclusion

Our analysis demonstrates that CO functions as a supplementary indicator of dysbiosis and potential environmental selector in the subgingival microbiome, creating conditions that favour the establishment and persistence of a distinct anaerobic microbial cluster dominated by *Fusobacterium* and *Prevotella*. This mechanistic understanding explains the clinical persistence of periodontal disease in smokers and provides a framework for developing targeted therapeutic interventions that address both microbial dysbiosis and environmental factors contributing to disease progression.

## Author Contributions

S.K., D.Hag. and S.K.B. contributed to formal analysis, validation, software and methodology. S.K., D.Hag. and K.P. contributed to investigation and data curation. B.E. and D.Har. contributed to project administration, conceptualisation and supervision. B.E., I.H. and D.Har. contributed to funding acquisition. P.E., K.L., T.‐S.K., T.K., J.Me., D.K., U.S. and B.E. contributed to resources. S.K., S.K.B. and D.Hag. contributed to writing – original preparation. J.Ma., J.Me., K.P., I.H., P.E., K.L., T.‐S.K., T.K., D.K., U.S., B.E. and D.Har. contributed to writing – review and editing.

## Conflicts of Interest

The authors declare no conflicts of interest.

## Supporting information


**Table S1:** Baseline variables stratified by smoking status.
**Table S2:** Generalized linear model results for the association of exhaled carbon‐monoxide (CO) with subgingival microbial dysbiosis.
**Table S3:** Fit statistics for three models at each study visit (A: CO only; B: Smoking only; C: CO+Smoking), adjusted for age, sex and treatment group.
**Table S4:** Zero‐inflated negative binomial mixed model regressing relative abundance of CO associated polymicrobial cluster on CO levels.

## Data Availability

The data that support the findings of this study are openly available in European Nucleotide Archive at http://www.ebi.ac.uk/ena/, reference number PRJEB51017.

## References

[jcpe70053-bib-0001] Al Kawas, S. , F. Al‐Marzooq , B. Rahman , et al. 2021. “The Impact of Smoking Different Tobacco Types on the Subgingival Microbiome and Periodontal Health: A Pilot Study.” Scientific Reports 11, no. 1: 1113. 10.1038/s41598-020-80937-3. 33441919 PMC7806658

[jcpe70053-bib-0002] Bergström, J. 2003. “Tobacco Smoking and Risk for Periodontal Disease.” Journal of Clinical Periodontology 30, no. 2: 107–113. 10.1034/j.1600-051X.2003.00272.x. 12622851

[jcpe70053-bib-0003] Bergström, J. , S. Eliasson , and J. Dock . 2000. “Exposure to Tobacco Smoking and Periodontal Health.” Journal of Clinical Periodontology 27, no. 1: 61–68. 10.1034/j.1600-051x.2000.027001061.x. 10674963

[jcpe70053-bib-0004] Bizzarro, S. , B. G. Loos , M. L. Laine , W. Crielaard , and E. Zaura . 2013. “Subgingival Microbiome in Smokers and Non‐Smokers in Periodontitis: An Exploratory Study Using Traditional Targeted Techniques and a Next‐Generation Sequencing.” Journal of Clinical Periodontology 40, no. 5: 483–492. 10.1111/jcpe.12087. 23489056

[jcpe70053-bib-0005] Callahan, B. J. , P. J. McMurdie , M. J. Rosen , A. W. Han , A. J. A. Johnson , and S. P. Holmes . 2016. “DADA2: High‐Resolution Sample Inference From Illumina Amplicon Data.” Nature Methods 13, no. 7: 581–583. 10.1038/nmeth.3869. 27214047 PMC4927377

[jcpe70053-bib-0006] Calsina, G. , J. Ramón , and J. Echeverría . 2002. “Effects of Smoking on Periodontal Tissues.” Journal of Clinical Periodontology 29, no. 8: 771–776. 10.1034/j.1600-051X.2002.290815.x. 12390575

[jcpe70053-bib-0007] Celik, D. , and A. Kantarci . 2021. “Vascular Changes and Hypoxia in Periodontal Disease as a Link to Systemic Complications.” Pathogens 10, no. 10: 1280. 10.3390/pathogens10101280. 34684229 PMC8541389

[jcpe70053-bib-0008] Chen, T. , W.‐H. Yu , J. Izard , O. V. Baranova , A. Lakshmanan , and F. E. Dewhirst . 2010. “The Human Oral Microbiome Database: A Web Accessible Resource for Investigating Oral Microbe Taxonomic and Genomic Information.” Database 2010: baq013. 10.1093/database/baq013. 20624719 PMC2911848

[jcpe70053-bib-0009] Coburn, R. F. , W. S. Blakemore , and R. E. Forster . 1963. “ENDOGENOUS CARBON MONOXIDE PRODUCTION IN MAN*.” Journal of Clinical Investigation 42, no. 7: 1172–1178. 10.1172/JCI104802. 14021853 PMC289385

[jcpe70053-bib-0010] Coretti, L. , M. Cuomo , E. Florio , et al. 2017. “Subgingival Dysbiosis in Smoker and Non‐Smoker Patients With Chronic Periodontitis.” Molecular Medicine Reports 15, no. 4: 2007–2014. 10.3892/mmr.2017.6269. 28260061 PMC5364964

[jcpe70053-bib-0011] Eickholz, P. , R. Koch , M. Göde , et al. 2023. “Clinical Benefits of Systemic Amoxicillin/Metronidazole May Depend on Periodontitis Stage and Grade: An Exploratory Sub‐Analysis of the ABPARO Trial.” Journal of Clinical Periodontology 50, no. 9: 1239–1252. 10.1111/jcpe.13838. 37293896

[jcpe70053-bib-0012] Eickholz, P. , R. Koch , T. Kocher , et al. 2019. “Clinical Benefits of Systemic Amoxicillin/Metronidazole May Depend on Periodontitis Severity and Patients' Age: An Exploratory Sub‐Analysis of the ABPARO Trial.” Journal of Clinical Periodontology 46, no. 4: 491–501. 10.1111/jcpe.13096. 30825384 PMC6594242

[jcpe70053-bib-0013] Eickholz, P. , K. Nickles , R. Koch , et al. 2016. “Is Furcation Involvement Affected by Adjunctive Systemic Amoxicillin Plus Metronidazole? A Clinical Trials Exploratory Subanalysis.” Journal of Clinical Periodontology 43, no. 10: 839–848. 10.1111/jcpe.12594. 27393928

[jcpe70053-bib-0014] Engel, R. R. , J. M. Matsen , S. S. Chapman , and S. Schwartz . 1972. “Carbon Monoxide Production From Heme Compounds by Bacteria.” Journal of Bacteriology 112, no. 3: 1310–1315. 10.1128/jb.112.3.1310-1315.1972. 4344922 PMC251565

[jcpe70053-bib-0015] Haffajee, A. D. , and S. S. Socransky . 2001. “Relationship of Cigarette Smoking to the Subgingival Microbiota.” Journal of Clinical Periodontology 28, no. 5: 377–388. 10.1034/j.1600-051x.2001.028005377.x. 11350499

[jcpe70053-bib-0016] Hagenfeld, D. , S. Kleine Bardenhorst , J. Matern , et al. 2023. “Long‐Term Changes in the Subgingival Microbiota in Patients With Stage III–IV Periodontitis Treated by Mechanical Therapy and Adjunctive Systemic Antibiotics: A Secondary Analysis of a Randomized Controlled Trial.” Journal of Clinical Periodontology 50, no. 8: 1101–1112. 10.1111/jcpe.13824. 37160709

[jcpe70053-bib-0017] Hagenfeld, D. , R. Koch , S. Jünemann , et al. 2018. “Do We Treat Our Patients or Rather Periodontal Microbes With Adjunctive Antibiotics in Periodontal Therapy? A 16S rDNA Microbial Community Analysis.” PLoS One 13, no. 4: e0195534. 10.1371/journal.pone.0195534. 29668720 PMC5906003

[jcpe70053-bib-0018] Hagenfeld, D. , J. Matern , K. Prior , et al. 2020. “Significant Short‐Term Shifts in the Microbiomes of Smokers With Periodontitis After Periodontal Therapy With Amoxicillin & Metronidazole as Revealed by 16S rDNA Amplicon Next Generation Sequencing.” Frontiers in Cellular and Infection Microbiology 10: 167. 10.3389/fcimb.2020.00167. 32477961 PMC7232543

[jcpe70053-bib-0019] Hagenfeld, D. , K. Prior , I. Harks , et al. 2019. “No Differences in Microbiome Changes Between Anti‐Adhesive and Antibacterial Ingredients in Toothpastes During Periodontal Therapy.” Journal of Periodontal Research 54, no. 4: 435–443. 10.1111/jre.12645. 30851050 PMC6767489

[jcpe70053-bib-0020] Hajishengallis, G. , and R. J. Lamont . 2021. “Polymicrobial Communities in Periodontal Disease: Their Quasi‐Organismal Nature and Dialogue With the Host.” Periodontology 2000 86, no. 1: 210–230. 10.1111/prd.12371. 33690950 PMC8957750

[jcpe70053-bib-0021] Harks, I. , D. Harmsen , M. Gravemeier , et al. 2014. “A Concept for Clinical Research Triggered by Suggestions From Systematic Reviews About Adjunctive Antibiotics.” Applied Clinical Research, Clinical Trials and Regulatory Affairs 1, no. 1: 43–50. 10.2174/2213476X01666140327211914.

[jcpe70053-bib-0022] Harks, I. , Y. Jockel‐Schneider , U. Schlagenhauf , et al. 2016. “Impact of the Daily Use of a Microcrystal Hydroxyapatite Dentifrice on De Novo Plaque Formation and Clinical/Microbiological Parameters of Periodontal Health. A Randomized Trial.” PLoS One 11, no. 7: e0160142. 10.1371/journal.pone.0160142. 27467683 PMC4965058

[jcpe70053-bib-0023] Harks, I. , R. Koch , P. Eickholz , et al. 2015. “Is Progression of Periodontitis Relevantly Influenced by Systemic Antibiotics? A Clinical Randomized Trial.” Journal of Clinical Periodontology 42, no. 9: 832–842. 10.1111/jcpe.12441. 26250060 PMC5054899

[jcpe70053-bib-0024] Hayashi, A. , H. Tauchi , and S. Hino . 1985. “Production of Carbon Monoxide by Bacteria of the Genera Proteus and Morganella.” Journal of General and Applied Microbiology 31, no. 3: 285–292. 10.2323/jgam.31.285.

[jcpe70053-bib-0025] Jarvis, M. J. , H. Tunstall‐Pedoe , C. Feyerabend , C. Vesey , and Y. Saloojee . 1987. “Comparison of Tests Used to Distinguish Smokers From Nonsmokers.” American Journal of Public Health 77, no. 11: 1435–1438. 10.2105/AJPH.77.11.1435. 3661797 PMC1647100

[jcpe70053-bib-0026] Javors, M. A. , J. P. Hatch , and R. J. Lamb . 2005. “Cut‐Off Levels for Breath Carbon Monoxide as a Marker for Cigarette Smoking.” Addiction 100, no. 2: 159–167. 10.1111/j.1360-0443.2004.00957.x. 15679745

[jcpe70053-bib-0027] Katayama, Y. A. , R. Kamikawa , and T. Yoshida . 2024. “Phylogenetic Diversity of Putative Nickel‐Containing Carbon Monoxide Dehydrogenase‐Encoding Prokaryotes in the Human Gut Microbiome.” Microbial Genomics 10, no. 8: 001285. 10.1099/mgen.0.001285. 39166974 PMC11338639

[jcpe70053-bib-0028] Kleine Bardenhorst, S. , D. Hagenfeld , J. Matern , et al. 2024. “The Role of the Oral Microbiota in the Causal Effect of Adjunctive Antibiotics on Clinical Outcomes in Stage III–IV Periodontitis Patients.” Microbiome 12, no. 1: 220. 10.1186/s40168-024-01945-3. 39462428 PMC11515798

[jcpe70053-bib-0029] Kotz, D. 2012. “Possible Reasons for Elevated Carbon Monoxide Levels in Self‐Reported ex‐Smokers.” Nicotine & Tobacco Research 14, no. 8: 900–901. 10.1093/ntr/ntr305. 22259146

[jcpe70053-bib-0030] Lee, J. , V. Taneja , and R. Vassallo . 2012. “Cigarette Smoking and Inflammation: Cellular and Molecular Mechanisms.” Journal of Dental Research 91, no. 2: 142–149. 10.1177/0022034511421200. 21876032 PMC3261116

[jcpe70053-bib-0031] Martin, M. 2011. “Cutadapt Removes Adapter Sequences From High‐Throughput Sequencing Reads.” EMBnet.Journal 17, no. 1: 10–12. 10.14806/ej.17.1.200.

[jcpe70053-bib-0032] McMurdie, P. J. , and S. Holmes . 2013. “Phyloseq: An R Package for Reproducible Interactive Analysis and Graphics of Microbiome Census Data.” PLoS One 8, no. 4: e61217. 10.1371/journal.pone.0061217. 23630581 PMC3632530

[jcpe70053-bib-0033] Mendes, S. S. , V. Miranda , and L. M. Saraiva . 2021. “Hydrogen Sulfide and Carbon Monoxide Tolerance in Bacteria.” Antioxidants 10, no. 5: 729. 10.3390/antiox10050729. 34063102 PMC8148161

[jcpe70053-bib-0034] Middleton, E. T. , and A. H. Morice . 2000. “Breath Carbon Monoxide as an Indication of Smoking Habit.” Chest 117, no. 3: 758–763. 10.1378/chest.117.3.758. 10713003

[jcpe70053-bib-0035] Moon, J.‐H. , J.‐H. Lee , and J.‐Y. Lee . 2015. “Subgingival Microbiome in Smokers and Non‐Smokers in Korean Chronic Periodontitis Patients.” Molecular Oral Microbiology 30, no. 3: 227–241. 10.1111/omi.12086. 25283067

[jcpe70053-bib-0036] O'Leary, T. J. , R. B. Drake , and J. E. Naylor . 1972. “The Plaque Control Record.” Journal of Periodontology 43, no. 1: 38. 10.1902/jop.1972.43.1.38. 4500182

[jcpe70053-bib-0037] Papapanou, P. N. , M. Sanz , N. Buduneli , et al. 2018. “Periodontitis: Consensus Report of Workgroup 2 of the 2017 World Workshop on the Classification of Periodontal and Peri‐Implant Diseases and Conditions: Classification and Case Definitions for Periodontitis.” Journal of Periodontology 89: S173–S182. 10.1002/JPER.17-0721. 29926951

[jcpe70053-bib-0038] Pihlstrom, B. L. , B. S. Michalowicz , and N. W. Johnson . 2005. “Periodontal diseases.” Lancet 366, no. 9499: 1809–1820. 10.1016/S0140-6736(05)67728-8. 16298220

[jcpe70053-bib-0039] R Development Core Team . 2017. R: A Language and Environment for Statistical Computing (3.4.3). R Foundation for Statistical Computing [Computer Software].

[jcpe70053-bib-0040] Rose, J. J. , L. Wang , Q. Xu , et al. 2017. “Carbon Monoxide Poisoning: Pathogenesis, Management, and Future Directions of Therapy.” American Journal of Respiratory and Critical Care Medicine 195, no. 5: 596–606. 10.1164/rccm.201606-1275CI. 27753502 PMC5363978

[jcpe70053-bib-0041] RStudio Team . 2016. RStudio: Integrated Development Environment for R. RStudio Inc. [Computer Software]. http://www.rstudio.com/.

[jcpe70053-bib-0042] Saberi Kakhki, K. C. L. , I. Harks , J. Matern , et al. 2025. “Association of Supragingival Plaque Management With Subgingival Microbiota Is Moderated by Adjunctive Antibiotics in Stage III‐IV Periodontitis Patients During Periodontal Therapy.” Journal of Oral Microbiology 17, no. 1: 2517043. 10.1080/20002297.2025.2517043. 40524743 PMC12168411

[jcpe70053-bib-0043] Sandberg, A. , C. M. Sköld , J. Grunewald , A. Eklund , and Å. M. Wheelock . 2011. “Assessing Recent Smoking Status by Measuring Exhaled Carbon Monoxide Levels.” PLoS One 6, no. 12: e28864. 10.1371/journal.pone.0028864. 22194931 PMC3241681

[jcpe70053-bib-0044] Song, L. , J. Li , X. Yuan , et al. 2017. “Carbon Monoxide‐Releasing Molecule Suppresses Inflammatory and Osteoclastogenic Cytokines in Nicotine‐ and Lipopolysaccharide‐Stimulated Human Periodontal Ligament Cells via the Heme Oxygenase‐1 Pathway.” International Journal of Molecular Medicine 40, no. 5: 1591–1601. 10.3892/ijmm.2017.3129. 28901402

[jcpe70053-bib-0045] Tamashiro, R. , L. Strange , K. Schnackenberg , et al. 2023. “Smoking‐Induced Subgingival Dysbiosis Precedes Clinical Signs of Periodontal Disease.” Scientific Reports 13, no. 1: 3755. 10.1038/s41598-023-30203-z. 36882425 PMC9992395

[jcpe70053-bib-0046] Wareham, L. K. , R. K. Poole , and M. Tinajero‐Trejo . 2015. “CO‐Releasing Metal Carbonyl Compounds as Antimicrobial Agents in the Post‐Antibiotic Era.” Journal of Biological Chemistry 290, no. 31: 18999–19007. 10.1074/jbc.R115.642926. 26055702 PMC4521022

[jcpe70053-bib-0047] Wirth, R. , G. Maróti , R. Mihók , et al. 2020. “A Case Study of Salivary Microbiome in Smokers and Non‐Smokers in Hungary: Analysis by Shotgun Metagenome Sequencing.” Journal of Oral Microbiology 12, no. 1: 1773067. 10.1080/20002297.2020.1773067. 32922678 PMC7448927

[jcpe70053-bib-0048] Wu, J. , B. A. Peters , C. Dominianni , et al. 2016. “Cigarette Smoking and the Oral Microbiome in a Large Study of American Adults.” ISME Journal 10, no. 10: 2435–2446. 10.1038/ismej.2016.37. 27015003 PMC5030690

