# Supplementary material for: Exhaled Carbon Monoxide Indicates Persistent Subgingival Dysbiosis After Periodontal Therapy
Source: J Clin Periodontol. 2025 Oct 16;53(2):170–8. doi: 10.1111/jcpe.70053 (PMC12803674; doi:10.1111/jcpe.70053)
Supplement: Supplementary file 1 — Table S1: Baseline variables stratified by smoking status. Table S2: Generalized linear model results for the association of exhaled carbon‐monoxide (CO) with subgingival microbial dysbiosis. Table S3: Fit statistics for three models at each study visit (A: CO only; B: Smoking only; C: CO+Smoking), adjusted for age, sex and treatment group. Table S4: Zero‐inflated negative binomial mixed model regressing relative abundance of CO associated polymicrobial cluster on CO levels. [file JCPE-53-170-s001.docx]

**Exhaled Carbon Monoxide as an Indicator of Subgingival Dysbiosis by promoting an anaerobic polymicrobial cluster during Periodontal Therapy - Supplementary Tables**

**Supplementary Table 1:** Baseline variables stratified by smoking status

|  | **Non-Smoker (n=94)** | **Smoker (n=69)** |
| --- | --- | --- |
| **Variable** | \| **n** \| **Mean (SD)** \| **Median (IQR)** \| **Min** \| **Max** \| **p-value** \| \| --- \| --- \| --- \| --- \| --- \| --- \| | \| **n** \| **Mean (SD)** \| **Median (IQR)** \| **Min** \| **Max** \| **p-value** \| \| --- \| --- \| --- \| --- \| --- \| --- \| |
| Centre  Berlin  Dresden  Frankfurt  Giessen  Greifswald  Heidelberg  Münster  Würzburg | 0.324   \| 11 \| 0.73 ± 1.19 \| 0.00 (1.00) \| 0.00 \| 3.00 \| \| --- \| --- \| --- \| --- \| --- \| \| 6 \| 0.67 ± 1.21 \| 0.00 (0.75) \| 0.00 \| 3.00 \| \| 6 \| 0.50 ± 0.55 \| 0.50 (1.00) \| 0.00 \| 1.00 \| \| 8 \| 1.62 ± 1.19 \| 2.00 (1.50) \| 0.00 \| 3.00 \| \| 5 \| 0.20 ± 0.45 \| 0.00 (0.00) \| 0.00 \| 1.00 \| \| 5 \| 0.80 ± 1.30 \| 0.00 (1.00) \| 0.00 \| 3.00 \| \| 32 \| 0.53 ± 0.92 \| 0.00 (1.00) \| 0.00 \| 4.00 \| \| 21 \| 0.71 ± 1.45 \| 0.00 (1.00) \| 0.00 \| 6.00 \|   (NA = 0) | 0.070   \| 10 \| 18.70 ± 13.12 \| 14.00 (6.25) \| 8.00 \| 51.00 \| \| --- \| --- \| --- \| --- \| --- \| \| 2 \| 10.50 ± 2.12 \| 10.50 (1.50) \| 9.00 \| 12.00 \| \| 6 \| 13.67 ± 2.16 \| 13.50 (2.50) \| 11.00 \| 17.00 \| \| 5 \| 19.60 ± 10.76 \| 17.00 (11.00) \| 11.00 \| 37.00 \| \| 6 \| 12.83 ± 6.40 \| 9.50 (9.25) \| 7.00 \| 21.00 \| \| 5 \| 11.00 ± 1.22 \| 11.00 (1.00) \| 9.00 \| 12.00 \| \| 19 \| 19.53 ± 9.58 \| 19.00 (9.50) \| 7.00 \| 42.00 \| \| 16 \| 17.88 ± 5.23 \| 17.00 (5.25) \| 11.00 \| 31.00 \|   (NA = 0) |
| Treatment group  Antibiotic  Placebo | 0.446   \| 46 \| 0.80 ± 1.26 \| 0.00 (1.00) \| 0.00 \| 6.00 \| \| --- \| --- \| --- \| --- \| --- \| \| 48 \| 0.58 ± 0.99 \| 0.00 (1.00) \| 0.00 \| 4.00 \|   (NA = 0) | 0.656   \| 35 \| 17.26 ± 9.93 \| 14.00 (8.00) \| 7.00 \| 51.00 \| \| --- \| --- \| --- \| --- \| --- \| \| 34 \| 16.85 ± 6.94 \| 16.50 (9.75) \| 7.00 \| 36.00 \|   (NA = 0) |
| Sex  Female  Male | 0.651   \| 45 \| 0.76 ± 1.35 \| 0.00 (1.00) \| 0.00 \| 6.00 \| \| --- \| --- \| --- \| --- \| --- \| \| 49 \| 0.63 ± 0.88 \| 0.00 (1.00) \| 0.00 \| 3.00 \|   (NA = 0) | 0.957   \| 38 \| 16.87 ± 8.60 \| 14.00 (8.75) \| 7.00 \| 51.00 \| \| --- \| --- \| --- \| --- \| --- \| \| 31 \| 17.29 ± 8.58 \| 16.00 (9.00) \| 7.00 \| 42.00 \|   (NA = 0) |
| Age  <45  45<55  >55 | **<0.01**   \| 11 \| 0.91 ± 1.22 \| 0.00 (1.50) \| 0.00 \| 3.00 \| \| --- \| --- \| --- \| --- \| --- \| \| 33 \| 1.18 ± 0.59 \| 1.00 (2.00) \| 0.00 \| 6.00 \| \| 50 \| 0.32 ± 1.49 \| 0.00 (0.75) \| 0.00 \| 2.00 \|   (NA = 0) | 0.299   \| 25 \| 18.56 ± 9.29 \| 16.00 (11.00) \| 8.00 \| 42.00 \| \| --- \| --- \| --- \| --- \| --- \| \| 32 \| 16.34 ± 6.52 \| 16.50 (7.25) \| 7.00 \| 37.00 \| \| 12 \| 15.83 ± 11.53 \| 12.00 (3.50) \| 9.00 \| 51.00 \|   (NA = 0) |
| Stage  III localized  III generalized  IV | 0.172   \| 15 \| 1.20 ± 1.70 \| 1.00 (1.50) \| 0.00 \| 6.00 \| \| --- \| --- \| --- \| --- \| --- \| \| 52 \| 0.73 ± 1.10 \| 0.00 (1.00) \| 0.00 \| 4.00 \| \| 27 \| 0.33 ± 0.55 \| 0.00 (1.00) \| 0.00 \| 2.00 \|   (NA = 0) | 0.526   \| 5 \| 15.00 ± 5.57 \| 12.00 (9.00) \| 10.00 \| 22.00 \| \| --- \| --- \| --- \| --- \| --- \| \| 38 \| 18.42 ± 9.73 \| 16.50 (10.50) \| 7.00 \| 51.00 \| \| 26 \| 15.46 ± 6.81 \| 13.50 (6.50) \| 9.00 \| 42.00 \|   (NA = 0) |
| Grade  B  C | 0.495   \| 21 \| 0.90 ± 1.22 \| 0.00 (1.00) \| 0.00 \| 4.00 \| \| --- \| --- \| --- \| --- \| --- \| \| 26 \| 0.65 ± 0.98 \| 0.00 (1.00) \| 0.00 \| 3.00 \|   (NA = 47) | 0.751   \| 4 \| 15.00 ± 5.23 \| 13.50 (6.50) \| 11.00 \| 22.00 \| \| --- \| --- \| --- \| --- \| --- \| \| 36 \| 16.31 ± 6.63 \| 15.00 (10.00) \| 7.00 \| 37.00 \|   (NA = 29) |
| O`Leary index Quantiles  Q1  Q2  Q3  Q4 | 0.601   \| 24 \| 0.88 ± 1.30 \| 0.00 (1.00) \| 0.00 \| 4.00 \| \| --- \| --- \| --- \| --- \| --- \| \| 24 \| 0.54 ± 1.28 \| 0.00 (1.00) \| 0.00 \| 6.00 \| \| 23 \| 0.57 ± 0.84 \| 0.00 (1.00) \| 0.00 \| 3.00 \| \| 23 \| 0.78 ± 1.04 \| 0.00 (1.50) \| 0.00 \| 3.00 \|   (NA = 0) | 0.403   \| 18 \| 20.28 ± 11.41 \| 19.50 (12.50) \| 8.00 \| 51.00 \| \| --- \| --- \| --- \| --- \| --- \| \| 17 \| 14.47 ± 4.17 \| 13.00 (7.00) \| 7.00 \| 22.00 \| \| 17 \| 16.82 ± 7.66 \| 16.00 (8.00) \| 9.00 \| 42.00 \| \| 17 \| 16.47 ± 8.69 \| 15.00 (6.00) \| 7.00 \| 37.00 \|   (NA = 0) |
| Amount PPD >/= 5mm % Quantiles  Q1  Q2  Q3  Q4 | 0.303   \| 24 \| 0.83 ± 1.49 \| 0.00 (1.00) \| 0.00 \| 6.00 \| \| --- \| --- \| --- \| --- \| --- \| \| 24 \| 0.75 ± 1.19 \| 0.00 (1.25) \| 0.00 \| 3.00 \| \| 23 \| 0.52 ± 0.73 \| 0.00 (1.00) \| 0.00 \| 2.00 \| \| 23 \| 0.65 ± 0.98 \| 0.00 (1.00) \| 0.00 \| 4.00 \|   (NA = 0) | 0.543   \| 18 \| 20.17 ± 10.90 \| 17.00 (9.75) \| 10.00 \| 51.00 \| \| --- \| --- \| --- \| --- \| --- \| \| 17 \| 16.00 ± 8.86 \| 12.00 (6.00) \| 7.00 \| 42.00 \| \| 17 \| 16.94 ± 6.34 \| 16.00 (10.00) \| 9.00 \| 31.00 \| \| 17 \| 14.94 ± 6.87 \| 13.00 (7.00) \| 7.00 \| 36.00 \|   (NA = 0) |

Smoking status: Assessed via self-report (questionnaire); Centre: Study centre in which the treatment and data collection was performed; Stage: Periodontitis stage according to 2018 classification; Grade: Periodontitis grade according to 2018 classification; O`Leary: O`Leary supragingival plaque index; O`Leary index Quantiles Q1-4 Non-Smoker: Q1: 3.75 - </= 18.75%, Q2: >18.75 - </= 33.62%, Q3: >33.62 - </= 57.81%, Q4: >57.81 – 100% ; O`Leary index Quantiles Q1-4 Smoker: Q1: 0.00 - </= 16.07%, Q2: >16.07 - </= 34.09%, Q3: >34.09 - </= 49.17%, Q4: >49.17 – 100%; Amount of PPD >/= 5mm: % of sites with pocket probing depths of 5mm or more; Amount PPD >/= 5mm% Quantiles Q1-4 Non-Smoker: Q1: 4.00 - </= 11.00%, Q2: >11.00 - </= 15.00%, Q3: >15.00 - </= 25.00%, Q4: >25 – 70% ; Amount PPD >/= 5mm% Quantiles Q1-4 Smoker: Q1: 3.00 - </= 11.00%, Q2: >11.00 - </= 19.00%, Q3: >19.00 - </= 35.00%, Q4: >35.00 – 78.00% ; n=number of subjects in the group; Median: Median of the CO-measurements indicated in ppm of the group at baseline visit; Min: Minimal CO measurement indicated in ppm of the Confounder-group at baseline visit; Max: Maximal CO measurement indicated in ppm of the Confounder-group at baseline visit

**Supplementary Table 2:** Generalized linear model results for the association of exhaled carbon-monoxide (CO) with subgingival microbial dysbiosis.

|  | **Before Therapy (V2)** | | | **2 months after therapy (V4)** | | | **14 months after therapy (V8)** | | | | **26 months after therapy (V12)** | | | |
| --- | --- | --- | --- | --- | --- | --- | --- | --- | --- | --- | --- | --- | --- | --- |
| *Predictors* | *Estimates* | *CI* | *p* | *Estimates* | *CI* | *p* | *Estimates* | *CI* | *p* | | *Estimates* | | *CI* | *p* |
| (Intercept) | 2.90 | 1.05 – 4.75 | **0.002** | -1.28 | -3.26 – 0.70 | 0.203 | 0.39 | -1.62 – 2.41 | | 0.700 | | 0.33 | -1.47 – 2.13 | 0.720 |
| Treatment Group  [antibiotic] | -0.19 | -1.49 – 1.12 | 0.777 | -5.85 | -7.26 – -4.44 | **<0.001** | -2.52 | -4.00 – -1.04 | | **0.001** | | -2.87 | -4.19 – -1.55 | **<0.001** |
| CO measurements ppm | 0.06 | -0.01 – 0.13 | 0.090 | 0.15 | 0.07 – 0.23 | **<0.001** | 0.12 | 0.03 – 0.20 | | **0.008** | | 0.09 | 0.01 – 0.17 | **0.025** |
| AGE [45<55] | -0.78 | -2.53 – 0.97 | 0.379 | 0.94 | -0.93 – 2.82 | 0.321 | -0.30 | -2.26 – 1.66 | | 0.760 | | 0.85 | -0.90 – 2.60 | 0.341 |
| AGE [>55] | -0.05 | -1.93 – 1.84 | 0.962 | 1.39 | -0.62 – 3.40 | 0.173 | -0.96 | -3.00 – 1.07 | | 0.352 | | 0.25 | -1.57 – 2.06 | 0.789 |
| SEX [male] | -0.69 | -1.99 – 0.61 | 0.295 | -0.24 | -1.65 – 1.16 | 0.731 | 0.95 | -0.54 – 2.44 | | 0.210 | | 0.54 | -0.79 – 1.88 | 0.423 |
| Observations | 163 | | | 159 | | | 163 | | | | 160 | | | |
| R^2^ / R^2^ adjusted | 0.035 / 0.005 | | | 0.337 / 0.315 | | | 0.122 / 0.094 | | | | 0.140 / 0.112 | | | |

Results of generalized linear model examining the association between exhaled carbon‐monoxide (CO) and subgingival microbial dysbiosis (as quantified in Kleine Bardenhorst et al. 2024), adjusting for age, sex, and treatment group. Analyses are stratified by study visit (V2, V4, V8, V12) to capture temporal variation in the CO–dysbiosis relationship. Coefficients (β) represent the change in dysbiosis score per 1-ppm increase in CO, with corresponding standard errors, 95% confidence intervals, and p-values.

**Supplementary Table 3:** Fit statistics for three models at each study visit (A: CO only; B: Smoking only; C: CO+Smoking), adjusted for age, sex and treatment group.

| **Visit** | **AICA** | **AICB** | **AICC** | **R²A** | **R²B** | **R²C** | **pANOVA A→C** | **Max VIFC** |
| --- | --- | --- | --- | --- | --- | --- | --- | --- |
| V2 | 718.0 | 714.0 | 715.0 | 0.0235 | 0.0498 | 0.0516 | 0.0332 | 1.8 |
| V4 | 748.0 | 753.0 | 738.0 | 0.345 | 0.390 | 0.392 | 0.0007 | 1.9 |
| V8 | 809.0 | 798.0 | 799.0 | 0.165 | 0.221 | 0.225 | 0.0006 | 1.7 |
| V12 | 775.0 | 778.0 | 766.0 | 0.164 | 0.215 | 0.221 | 0.0010 | 1.9 |

Model comparison based on Akaike Information Criterion (AIC; lower = better; bold indicates best among models A–C) and coefficient of determination (R²; variance explained; higher = better; bold indicates best). The p-value for partial analysis of variance (pANOVA; comparison A → C) tests whether adding smoking status to the carbon monoxide (CO)-only model significantly improves model fit. Max VIFC refers to the maximum variance inflation factor in Model C, with values below 2 indicating minimal collinearity.

**Supplementary Table 4:** Zero-inflated negative binomial mixed model regressing relative abundance of CO associated polymicrobial cluster on CO levels.

|  | **Polymicrobial Cluster** | |
| --- | --- | --- |
| *Predictors* | *Rate Ratios* | *CI (95%)* |
| Intercept | 0.20 | 0.16 – 0.25 |
| **CO concentration (ppm/10)** | **1.16** | **1.08 – 1.26** |
| Age: 45<55 | 0.96 | 0.79 – 1.15 |
| Age: >55 | 0.97 | 0.79 – 1.18 |
| Sex: male | 1.03 | 0.89 – 1.20 |
| PPD% | 0.99 | 0.99 – 1.00 |
| O’Leary Index | 1.00 | 1.00 – 1.00 |
| Antibiotic Treatment | 1.11 | 0.95 – 1.20 |

The model is adjusted for age, sex, pocket probing depth and supragingival plaque as measured via the O’Leary Index and antibiotic treatment. A random intercept for subject was included to account for repeated measurements.
